# Effects of Oral Anticoagulant Therapy on Gene Expression in Crosstalk between Osteogenic Progenitor Cells and Endothelial Cells

**DOI:** 10.3390/jcm8030329

**Published:** 2019-03-08

**Authors:** Luca Dalle Carbonare, Monica Mottes, Anna Brunelli, Michela Deiana, Samuele Cheri, Silvia Suardi, Maria Teresa Valenti

**Affiliations:** 1 Department of Medicine, University of Verona, Pz.le Scuro 10, 37134 Verona, Italy; luca.dallecarbonare@univr.it (L.D.C.); anna.brunelli@univr.it (A.B.); michela.deiana@univr.it (M.D.); samuele.cheri@univr.it (S.C.); silvia.suardi@univr.it (S.S.); mariateresa.valenti@univr.it (M.T.V.); 2 Department of Neurosciences, Biomedicine and Movement Sciences, University of Verona, Strada Le Grazie 8, 37134 Verona, Italy

**Keywords:** anticoagulants, circulating progenitors, osteogenesis, gene expression

## Abstract

Direct oral anti-coagulants (DOACs) are employed in clinical practice for the prevention and treatment of recurrent venous thromboembolism and for the prevention of stroke in non-valvular atrial fibrillation. DOACs directly and reversibly inhibit activated factor X or thrombin and can interfere with other pathophysiological processes such as inflammation, lipid metabolism, and bone turnover. We aimed to evaluate the possible effects of DOACs on osteogenesis and angiogenesis. We treated 34 patients affected by cardiovascular disorders with DOACs; biochemical and molecular analyses were performed before and after three months of treatment. Circulating progenitors (CPs; CD34^−^, CD45^−^, CD14^−^, CD73^+^, CD105^+^), which share typical bone marrow stem cell (MSCs) features, were harvested from peripheral blood of the study subjects to monitor the expression of osteogenesis-related genes *RUNX2* and *SPARC*. Human umbilical vein endothelial cells (HUVECs) were used to probe angiogenesis-related *VEGF*, *CD31*, and *CD105* gene expression. We performed co-culture experiments using a commercial human mesenchymal stem cells line (hMSCs) obtained from bone marrow and HUVECs. Clinical parameters related to bone metabolism, coagulation, renal and liver function, and the lipid profile were evaluated. Values of the C-terminal telopeptide type I collagen (CTX) increased after the treatment. We found a significant increase in osteogenesis marker gene expression in CPs after three months of anticoagulant therapy. An increase in the *RUNX2* expression determinant alone was detected instead in hMSCs co-cultured with HUVECs in the presence of treated patients’ sera. The VEGF, CD31, and CD105 marker genes appeared to be significantly upregulated in HUVECs co-cultured with hMSCs in the presence of treated patients’ sera. Under these conditions, new vessel formation increased as well. Our results highlight an unexpected influence of DOAC therapy on osteogenic commitment and vascular endothelial function promotion.

## 1. Introduction

Direct oral anti-coagulants (DOACs) are direct and selective inhibitors of specific factors of the coagulation cascade [1]. Those currently in use are three activated factor X (AFX) inhibitors, rivaroxaban, apixaban, and edoxaban, and a thrombin inhibitor, dabigatran. DOACs are often referred to as non-vitamin K oral anti-coagulants (NOACs) to emphasize their distinction from vitamin K antagonists (VKAs), whose main exponent is warfarin. The VKA mechanism of action in the coagulation cascade is based on antagonizing vitamin K, an essential cofactor for γ-carboxylation of factors II, VII, IX, and X [2].

DOACs are indicated for the prevention and treatment of recurrent venous thromboembolism (VTE) and for the prevention of stroke and systemic embolization in patients with non-valvular atrial fibrillation (NVAF). Patients who underwent mechanical valve prostheses or suffered from moderate to severe mitral stenosis are excluded from the treatment [2,3]. Thromboembolic disease can occur both in the arterial and the venous system with different characteristics in terms of the determining factors, thrombus morphology, and associated diseases.

Factor Xa and thrombin have been shown to play fundamental roles in coagulation and in other processes, mainly through the activation of protease-activated receptor (PARs), a family of proteolytic cleavage-activated receptors found in almost all tissues and cell types [4]. Thrombin-PAR-1 interaction activates the signaling axis involved in actinic cytoskeleton modifications of endothelial cells, thus contributing to endothelial barrier disruption and increased permeability [5]. In patients suffering from vascular disease, factor Xa modifies the expression levels of oxidative stress-induced proteins, thereby affecting aerobic mitochondrial metabolism [6]. Through the stimulation of PAR-1 and -2 and the activation of insulin-like growth factor binding protein 5 (IGFBP5) and p53, factor Xa induces a senescent phenotype and the expression of pro-inflammatory molecules in the smooth muscle cells of the vascular wall [7]. Due to their key role in various signaling pathways in both vascular and immune systems, factor Xa and thrombin appear to participate in atherosclerosis and plaque progression as well as in remodeling and fibrosis processes [7].

Consequently, the ability of DOACs to act as inhibitors of these non-hemostatic functions of factor Xa and thrombin has been tested. The role of rivaroxaban in promoting angiogenesis has been demonstrated. Increased levels of vascular endothelial growth factor (VEGF) and nitric oxide synthase (eNOS) were detected in animal models in response to conditions of relative tissue ischemia [8]. Apixaban also appears to influence vasomotor function by increasing vasodilation through PAR-2-induced NO synthesis [9]. Various studies in animal models seem to confirm that DOACs attenuate endothelium interactions with leukocytes and platelets, which represent the first step in the inflammatory and atherothrombotic process in microcirculation [10]. The same stabilizing effect on atherosclerotic lesions and inhibition of vascular pathology progression have been documented in ApoE knockout mice, characterized by high levels of very low density lipoprotein (VLDL) and low density lipoprotein (LDL). A reduction in plaque size and inherent oxidative and inflammatory phenomena with a concomitant reduction of nuclear factor kappa-light-chain-enhancer of activated B cells (NF-κB), matrix metallopeptidase 9 (MMP-9), vascular cell adhesion protein 1 (VCAM-1), and tumor necrosis factor α (TNFα) were reported [10]. In vitro treatment of endothelial progenitor cells with an activated factor X inhibitor increased the activity of eNOS and the expression of key angiogenic growth factors, such as VEGF. Microscopic observations showed the increased ability of endothelial progenitors to produce vascular tubular structures, promoting endothelial cell functions in terms of repair and regeneration and possible pro-angiogenic properties derived from the inhibition of factor Xa [8].

Modulation of human mesenchymal stem cells via crosstalk with endothelial cells was demonstrated by Bidarra et al. [11], who demonstrated that human mesenchymal stem cells (hMSCs) co-cultivation with human umbilical vein endothelial cells (HUVECs) increased proliferation and osteogenic differentiation. Therefore, osteogenesis and angiogenesis are strongly related processes [12]. *RUNX2* knock out has been associated with reduced VEGF synthesis and impaired vascular invasion during cartilage differentiation [13]. RUNX2 transcription factor is present in endothelial cells as well as in vascular smooth muscle cells during in vivo angiogenesis [14,15]. Therefore, on the basis of pleiotropic effects and considering that osteogenesis and angiogenesis are related processes, we hypothesized that DOACs might interfere with bone formation.

To gain a more in-depth knowledge of anticoagulant treatment effects on bone and vasculature, we evaluated the modulation of gene expression profiles induced by DOACs in circulating progenitor cells. We analyzed the effects of crosstalk between endothelial cells and marrow stem cells (MSCs) in the presence of sera collected from patients during the treatment with DOACs.

## 2. Experimental Section

### 2.1. Subjects

The study was conducted at Verona University Hospital, Italy. We recruited 34 patients with a mean age of 79 ± 9 years from January to June 2018. Of the 34 patients, 23 were sourced from the Department of Internal Medicine for Atherothrombotic and Degenerative Diseases, and 11 patients were selected by the Stroke Unit. Written informed consent was obtained from all participants, and the study was approved by the Ethical Committee of Azienda Ospedaliera Universitaria Integrata of Verona, Italy (No. 1538).

Of the 34 enrolled, 18 patients presented a previous diagnosis of non-valvular atrial fibrillation (NVAF), 8 patients were under observation after the first detection of deep vein thrombosis (DTV) of the lower limbs or pulmonary embolism (PE). The last group of 8 patients was diagnosed with ischemic stroke. Among these, a diagnosis of NVAF, previously unknown, was confirmed in 3 patients during the investigations to attest to the cardioembolic etiology of the ischemic stroke that had led to hospitalization.

A summary of the previously assumed therapy, classifying patients according to the underlying disease, is provided in Table 1.

A 3-month follow-up was scheduled for all patients enrolled in the study.

### 2.2. Biochemical Parameters

At the beginning of direct oral anticoagulant therapy and after 3 months, the following fasting blood parameters were evaluated: blood count, coagulation, lipid profile, renal and liver function, and phospho-calcic parameters. We considered the hemoglobin concentration, hematocrit, platelet count, prothrombin time- international normalized ratio (PT-INR), activated partial thromboplastin time (aPTT), total cholesterol, high density lipoprotein (HDL), triglycerides, creatinine, aspartate tansaminase (AST), alanine transaminase (ALT), calcemia, C-terminal telopeptide type I collagen (CTX), and vitamin D concentration. Using the Cockroft–Gault formula, the glomerular filtration rate (eGFR) was estimated, and the serum LDL fasting serum was calculated using the Friedwald formula.

### 2.3. Isolation of Circulating Progenitor Cells

Before and after 3 months of DOAC treatment, each patient underwent peripheral venous blood sampling, and CPs—cells with typical bone MSCs features—were collected, as reported previously [16]. CPs were isolated from 25 mL of heparinized blood by a depletion method, including two Ficoll procedures to remove hematopoietic cells using the antibodies cocktail. Firstly, peripheral blood mononuclear cells (PBMCs) were obtained by a gradient centrifugation at 800× *g* for 30 min at 20 °C (first Ficoll procedure). Then, to remove unwanted hematopoietic cells, a Rosette-Sep antibody cocktail was used with 5 mL of whole blood mixed with the PBMCs obtained by the first Ficoll; the antibody cocktail was incubated with samples for 20 min at room temperature. Then, a second Ficoll procedure was performed to remove the unwanted CD3, CD14, CD19, CD38, and CD66b positive cells crosslinked to red blood cells (glycophorin A).

Generally, CPs originating osteogenic, condrogenic, and adipogenic cells are defined as CD34^−^, CD45^−^, CD14^−^, CD73^+^, CD105^+^ cells [17,18]. Therefore, we evaluated their phenotype by analyzing gene expression for CD3, CD14, CD19, CD45,CD34, CD73, and CD105 markers, as reported previously [19]. This method allows the analysis of the phenotype of cells isolated by stringent purification strategies, as previously described [20].

### 2.4. RNA Extraction and Reverse Transcription

RNA was extracted using the Q RNA Assay Mini-Kit (Quiagen, Hilden, Germany) with DNase I treatment. The obtained RNA was quantified by measuring absorbance at 260 nm, and the purity was checked by measuring the 260/280 absorbance ratio. First-strand complementary DNA (c-DNA) synthesis was performed using the First Strand cDNA Synthesis Kit (GE Healthcare, Little Chalfont, UK) using random hexamers (GE Healthcare, Little Chalfont, UK) according to the manufacturer’s protocol. The product was then aliquoted in equal volumes and stored at −80 °C until use.

### 2.5. Real Time PCR (RT-PCR)

PCR was performed using Taqman Universal PCR Master mix (Thermofisher Corporation, Waltham, MA, USA). Pre-designed gene-specific primers and probe sets for each gene (osteogenic gene markers: *RUNX2*, Hs00231692_m1; *SPARC*, Hs00234160_m1; endothelial gene markers; *VEGF*, Hs00900055_m1; *CD31*, Hs01065279_m1; *CD105*, Hs00923996_m1; hematopoietic gene markers: *CD3*, Hs00174158_m1; *CD14*, Hs02621496-s1; *CD19*, Hs00174333_m1; *CD45*, Hs00174541_m1; *CD34*, HS00156373_m1; mesenchymal gene marker *CD73*, Hs00159686_m1: *CD105*, Hs00923996_m1 in *CD34* negative cells; and housekeeping genes: *ACTB*, 4326315E, and *B2M*, 4326319E) were obtained from Assay-on-Demand Gene Expression Products (Thermofisher Corporation, Waltham, MA, USA). Real-time (RT) amplifications included 10 min at 95 °C (AmpliTaq Gold activation), followed by 40 cycles at 95 °C for 15 seconds, and finally at 60 °C for 1 min. Thermocycling and signal detection were performed with an ABI Prism 7300 Sequence Detector (Applied Biosystems; Foster City, CA, USA). Ct values for each reaction were determined using TaqMan SDS analysis software (Applied Biosystems; Foster City, CA, USA), as reported previously [19]. The analyses were performed by using the 2−ΔΔCT method. This method allows for the calculation of the relative gene expression levels between different samples [21].

### 2.6. Cell Cultures and Co-Cultures Preparation

Bone marrow-hMSCs (Heidelberg, Germany) were cultured and induced to proliferate in MSC growth medium (MSCGM; Cologne, Germany). The osteosarcoma cell line MG63 and normal human epidermal melanocytes (NHEM) (used as low expressing cells) were purchased from American Type Culture Collection (ATCC CTRL-1619TM; Rockville, MD, USA) and cultured in the presence of RPMI medium. Cell lines were cultured under a humidified atmosphere of 5% CO_2_ in the presence of growth medium (MSCGM or RMPI 1640 (Sigma-Aldrich; St. Louis, MO, USA) containing 10% fetal bovine serum (FBS; Sigma-Aldrich; St. Louis, MO, USA) and supplemented with antibiotics (1% penicillin/streptomycin) and 1% glutamine.

Primary HUVEC at passage 10 was provided from Professor Bazzoni of the University of Verona, Italy. Untreated cells were used as the positive control. Cells were cultured in a 75 cm^2^ culture flask coated with Matrigel (Corning Incorporated; New York, NY, USA) in the presence of M200 medium and 10% low serum growth supplement (LSGS) (Thermo Fisher Scientific, Waltham, MA, USA) under a humidified atmosphere of 5% CO_2_. Co-cultured cells were arranged in a 6-well Transwell plate with 0.4 µm pores (Corning Incorporated; Corning, NY, USA). HUVECs were cultured in the presence of 10% sera from patients enrolled in the study. Sera were obtained from 6 mL of peripheral venous blood by centrifugation and stored at −80 °C until use. Co-cultures of MSCs and HUVECs with a 1:1 ratio were prepared and treated in the presence of 10% of sera collected before and after 3 months of treatment.

### 2.7. Immunofluorescence

Transwell membranes were cut using a scalpel and washed three times in 1× phosphate buffered saline (PBS). Membranes were incubated for 1 h at room temperature with 10% goat serum. *RUNX2* antibody (Novusbio, Centennial, CO, USA) at 1:100 dilution was incubated in agitation overnight at room temperature. For signal detection, Alexa Fluor 488 (Life Technologies, Carlsbad, CA, USA) and ProLong™ Gold Antifade Mountant with 4’,6-diamidin-2-fenilindolo (DAPI) for nuclei counterstaining (Life Technologies, Carlsbad, CA, USA) were used. Photos were acquired using a Leica DM2500 microscope (Leica Microsystems, Wetzlar, Germany) at 10, 20, 40, and 63× magnifications.

### 2.8. Alizarin Staining

After 21 days of culture in specific MSCGM medium, cells were fixed with 70% ethanol and subsequently rinsed with deionized water. Cells were then treated for 5 min with 40 mM Alizarin red S at pH 4.1 and again washed with PBS for 15 min. For staining technique control, we used MG63 cells. MG63 cells cultured with 125 μM of ascorbic acid were employed as the positive staining control for calcium deposition. We used undifferentiated hMSCs as the negative control for calcium deposition.

### 2.9. Net-Like Formation Assay

Six-well Transwell plates with 0.4 μm pores (Corning Incorporated, Corning, NY, USA) were used. HUVEC cells were plated in the chamber, and MSC cells were plated in the inserts and cultured with their specific culture medium at a 1:1 ratio, both in duplicate. After 7 days, the inserts were removed and stained with Mayer’s hematoxylin (Sigma Aldrich; St. Louis, MO, USA) for 1 min and photographed at 5× magnification using a Leica DMi1 microscope (Leica Microsystems, Wetzlar, Germany). Completely enclosed spaces were considered to quantify net-like structures, as previously reported [22].

### 2.10. Statistical Analysis

All data were normally distributed and are expressed as mean ± standard deviation. Differences between groups were tested for significance using Student’s *T*-test and multiple ANOVA measurements. Statistical significance was considered for a *p*-value of <0.05. Statistical analyses were performed using SPSS for Windows, version 22.0 (SPSS Inc., Chicago, IL, USA).

## 3. Results

### 3.1. Clinical and Biochemical Data

Table 2 shows the baseline anthropometric and biochemical characteristics of the patients enrolled in the study compared to those observed three months after the beginning of direct oral anticoagulant therapy.

A significant increase in CTX emerged after three months of treatment with DOACs (CTX at first visit: 0.31 ± 0.09 ng/mL; at three months: 0.36 ± 0.12 ng/mL; *p* < 0.05), if it remained in the normal range. No statistically significant changes in the other biochemical parameters examined were observed.

On average, after three months of treatment with DOACs, hemoglobin levels tended to decrease. The result is not statistically significant and can be traced to the effect of anticoagulant therapy. None of patients experienced bleeding or other complications related to anticoagulant treatment. Data collected showed PT-INR levels above the normal range at the end of the observational period but still lower than the initial values. PT-INR values at first visit were influenced by the fact that 16 of the 34 selected patients previously were treated with warfarin.

Notably, there was an improvement in vitamin D levels measured at the follow-up visit (vitamin D at first visit: 17 ± 7 ng/mL; at three months: 25 ± 14 ng/mL), but levels remained slightly low. All patients in whom hypovitaminosis D was initially found were given the indication to perform supplementation with cholecalciferol 50,000 U.I./month.

To assess the role of the underlying disease, we also tested for the presence of any differences in blood parameters at baseline and after three months in patients diagnosed with NVAF compared with those with VTE or ischemic stroke. A significantly higher level of baseline PT-INR was observed in patients receiving direct oral anticoagulant therapy for NVAF compared with those presenting acute thromboembolism or cerebral ischemic event (Table 3). These data indicate a trend in preferring DOACs for patients diagnosed with thromboembolism or acute ischemic stroke. Warfarin still seems to be the first choice in clinical practice for patients with NVAF. At three months, no statistically significant difference could be seen between groups of patients with different underlying diseases.

### 3.2. DOACs Enhance Osteogenic Commitment

The percentage of CD105^+^ and CD73^+^ cells was higher that 60% with low expression of CD14, CD45, and CD34 markers. CPs collected before and after three months of treatment showed the same cluster differentiation markers expression (Table 4).

**Figure 1:**
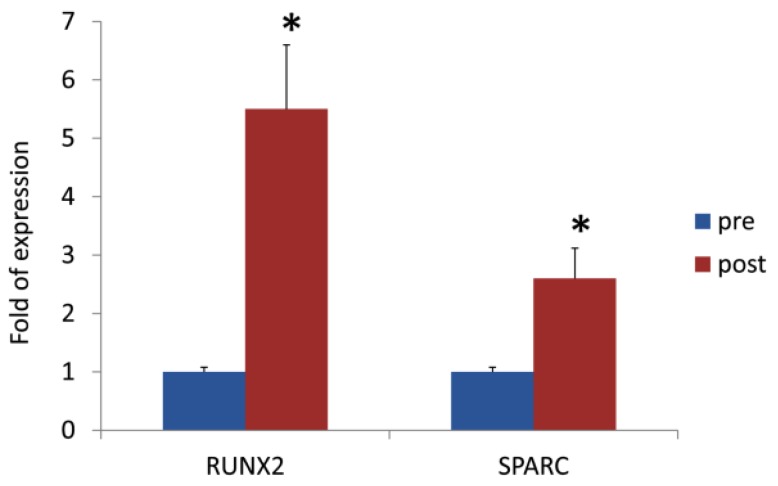
*RUNX2* and *SPARC* gene expression in circulating progenitors before and after three months of DOAC treatment. * *p* < 0.05.

The analysis of the osteogenic gene expression in CPs demonstrated a significant increase in the expression of *RUNX2* (5.5-fold increase, *p* < 0.05) and *SPARC* (2.6-fold increase, *p* < 0.05) three months after the beginning of direct oral anticoagulant therapy (Figure 1).

**Figure 2:**
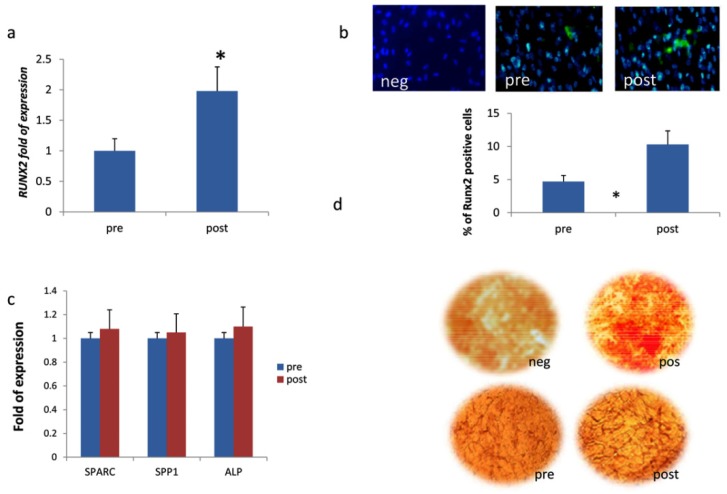
(**a**) *RUNX2* gene expression and (**b**) protein expression in hMSCs co-cultured with human umbilical vein endothelial cells (HUVEC) and patients’ sera before (pre) and after (post) three months of DOAC treatment. (**c**) *SPARC*, *SPP1,* and *ALP* expression and (**d**) Alizarin red staining in the same setting of cells to evaluate osteogenic maturation; neg [wo *RUNX2* antobody; (**b**)] [undifferentiated hMSC; (**d**)]; pos (ascorbic acid treated-MG63 cells). Magnification in (**b**) 10 × and (**d**) 4×. * *p* < 0.05.

Expression of osteogenic genes was analyzed in MSCs when co-cultured with HUVEC and in the presence of patients’ sera before and after three months of DOAC treatment. We observed a higher level of mRNA (Figure 2A) and protein (Figure 2B) expression of transcription factor *RUNX2* in the presence of patients’ sera after treatment. The expression of genes related to osteoblastic maturation, such as *SPARC*, *ALP*, and *SPP1* (Figure 2C), as well as the calcium deposition evaluated using Alizarin red staining (Figure 2D), did not change.

### 3.3. DOACs Increase Expression of Key Angiogenic Markers in Presence of MSCs

**Figure 3:**
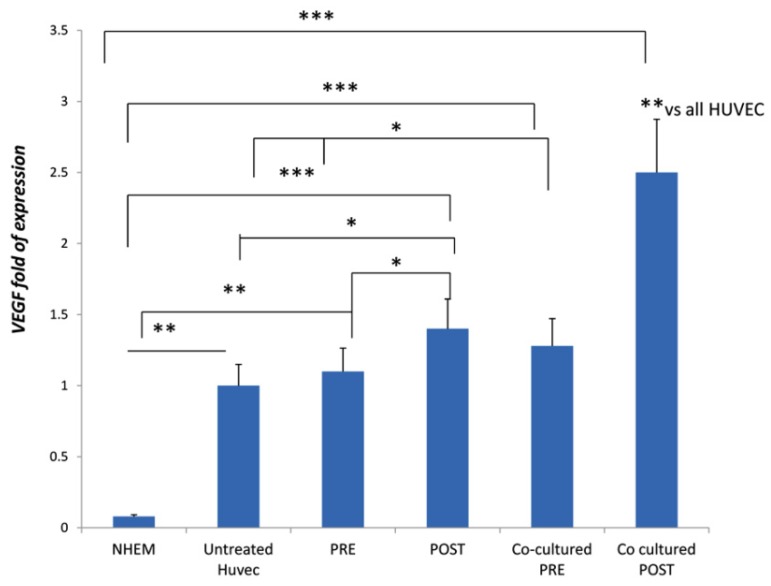
*VEGF* expressed in HUVEC treated with patients’ sera collected before (PRE) and after (POST) three months of DOAC treatment. The sera (PRE or POST treatment) were added in HUVEC co-cultured with hMSCs. NHEM: normal melanocytes used as low expressing cells. * *p* < 0.05; ** *p* < 0.01; *** *p* < 0.005.

In HUVECs cultured in the presence of sera collected from patients enrolled in the study, the gene expressions of *VEGF*, *CD31*, and *CD105* were analyzed. As we analyzed the relative gene expression by using the 2−ΔΔCT method, we considered the untreated HUVECs as the calibrator (baseline levels; value 1). We compared the expression levels of all samples with a low-level expressing cell line (the normal melanocytes cell line NHEM). Compared with baseline levels, *VEGF* marker expression increased when cultured with sera of patients after three months of direct oral anticoagulation treatment (Figure 3). However, VEGF expression was higher in HUVEC co-cultured with MSCs and in the presence of sera from patients after the treatment (Figure 3).

**Figure 4:**
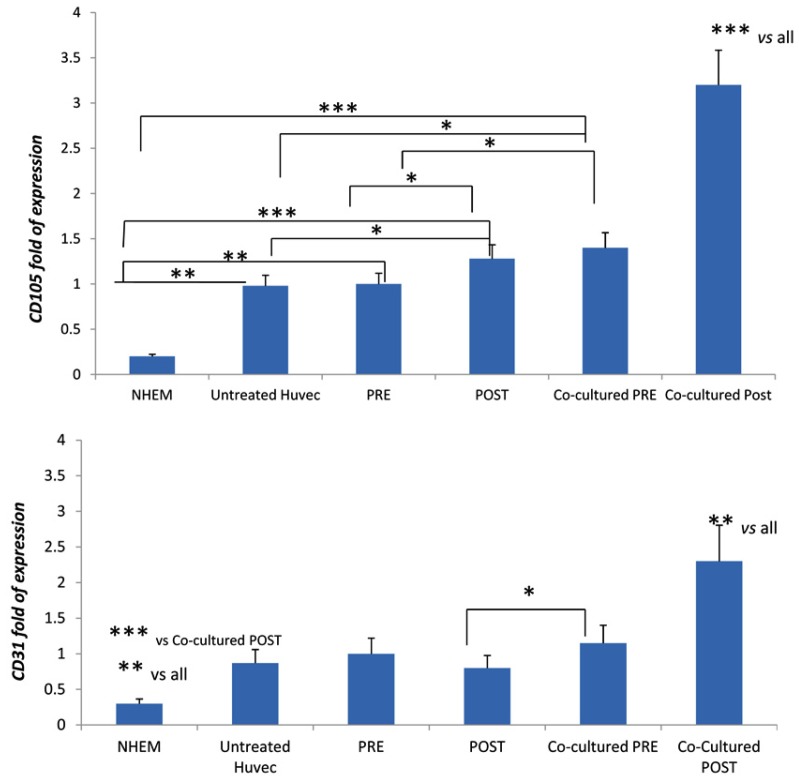
CD105 and CD31 markers; expression in HUVECs in the presence of sera collected from patients before (pre) and after (post) three months of DOAC treatment. The sera (pre or post treatment) were added in HUVEC co-cultured with MSCs. NHEM: normal melanocytes used as low expressing cells. * *p* < 0.05; ** *p* < 0.01; *** *p* < 0.005.

Similarly, the expression of *CD105* increased in HUVEC in the sera of patients after three months of treatment (Figure 4); this effect was more remarkable in HUVEC co-cultured with MSCs (Figure 4). The expression of CD31 was higher in HUVEC in the presence of sera of patients after three months of treatment only when co-cultured with MSCs (Figure 4).

**Figure 5:**
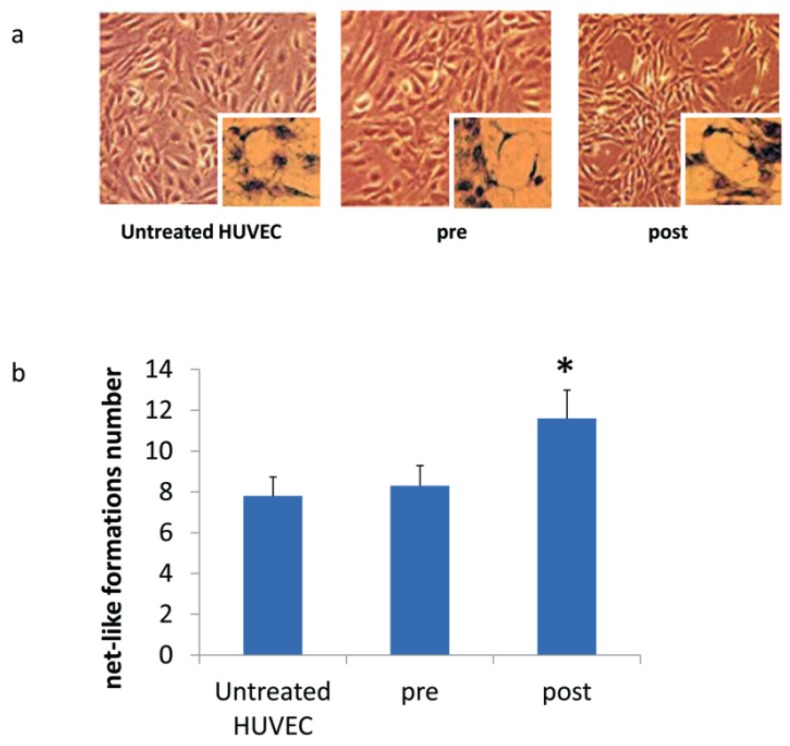
(**a**) Net-like formations originated from HUVECs co-cultured with hMSCs without (no sera added to the medium; untreated HUVEC) or with sera collected from patients before (pre) and after three months of DOAC treatment (post). Magnification 10×; insert magnification 40×; (**b**) the graph shows the net-like formations number calculated on the above samples.

Enhanced angiogenesis after three months of treatment was confirmed by the increased number of net-like formations when HUVECs were co-cultured with sera of patients (Figure 5).

## 4. Discussion

Growing interest is emerging in the supposed involvement of DOACs in regulating vascular endothelial function.

In HUVEC cultures and animal models, factor Xa appears to be characterized by anti-angiogenic properties, mediated by the activation of PAR-1, and supported by endothelium stabilizing factors secreted by pericytes, fibroblasts, and the smooth muscle cells of the vessel wall.

With regard to endothelial function, the data in the present study are in line with previous evidence. In HUVECs treated with the sera of the enrolled patients three months after the beginning of DOACs therapy, we observed increased expression of *VEGF* and *CD105* markers, which are involved in the processes of vascular renewal and the maintenance of vessel wall integrity. Vascular endothelial growth factor (VEGF) family members are involved in angiogenesis, having VEGFR as their specific tyrosine-kinase receptor located on the cell surface [23]. CD105, also known as endoglin, is part of the TGF-β receptor complex. The absence of endoglin does not affect the formation of the first endothelial networks (mediated by VEGF), but endoglin is essential for the efficient organization of mature vascular structures [23]. Expression of the CD31 marker, conversely, did not change in HUVECs treated with patients’ sera after three months of DOACs. CD31, encoded by the *PECAM1* gene, is normally expressed by endothelial cells and contributes to the maintenance of the permeability barrier [24]. However, expression levels of *VEGF*, *CD105*, and *CD31* markers increased considerably when HUVECs were co-cultured with MSCs. These findings demonstrate that DOACs improve endothelial function by acting on HUVECs-MSCs crosstalk. Importantly, MSCs have been shown to release several exosomes containing microRNAs (miRNAs), which can be transferred to endothelial cells, promoting angiogenesis [25].

With respect to the role of anticoagulants in the processes of mineralization and vascular calcification, the mechanisms linked to the inhibition of vitamin K by VKAs are well known [26]. In osteoblastic cell cultures, warfarin was confirmed to inhibit the differentiation process through the reduction of *alkaline phosphatase (ALP)*, *Collagen 1A1 (COL1A1)*, *Osteocalcin*, *Osterix*, genes expression, and the interruption of *RUNX2* transcriptional activity [27]. VKAs treatment could therefore result in an increased risk of osteoporosis, vascular calcification, and blockage of vessels renewal [28]. Comparing the effects of thrombus prophylaxis with various anticoagulant drugs, enoxaparin and warfarin tend to inhibit gene expression of osteogenic differentiation markers, whereas the FXa inhibitor rivaroxaban, by acting on IGF2, promotes osteoblastic survival. Our data confirm the effects of rivaroxaban in promoting osteogenic commitment. Analyzing *RUNX2* and SPARC expression in circulating progenitors, a statistically significant increase was observed at the end of the study. Therefore, even if additional studies are needed, our data suggest an influence of DOACs in regulating bone forming processes; this aspect could suggest a further distinction from VKAs. After three months of treatment with DOACs, a significant increase in CTX was observed in patients, although within the normal range. Therefore, we suppose that DOACs promote bone resorption as well. Bone formation and resorption are coupled processes. Further studies should investigate skeletal system-related molecular pathways affected by DOACs.

Our data regarding increased *RUNX2* gene expression were confirmed in co-cultures of MSCs and HUVECs treated in the presence of sera collected from patients after DOAC treatment. However, despite an increased expression of *RUNX2*, the expression of genes such as *SPARC*, *SPP1*, and *ALP*, which are related to osteogenic maturation, did not change.

The increased expression of *RUNX2* uncoupled from a subsequent upregulation of the osteogenic maturation markers suggests that MSCs co-cultured with HUVECs are halted in an undifferentiated state by DOACs. This is an important finding since it suggests how the calcification process may be avoided when MSCs are tightly in contact with endothelial cells.

## 5. Conclusions

Our results suggest a possible influence of direct oral anticoagulant therapy not only on the coagulation system but also on bone metabolism and the endothelial and restorative functions of the vessel wall. The latter is of particular interest, as DOAC-treated pathological conditions include endothelial dysfunction, alteration of the hemostatic balance, and hypercoagulability. Further studies on a larger patient sample for a longer observational period might confirm the specific properties of DOACs, suggesting new perspectives for their targeted use.

## Figures and Tables

**Table 1:** Previously prescribed therapies in patients classified according to the underlying disease. The largest group of patients reported warfarin treatment for NVAF. NVAF non-valvular atrial fibrillation, VTE venous thromboembolism, ASA acetylsalicylic acid.

Disease	Previous Therapy	No. of Patients
NVAF	No treatment	0
	Warfarin	12
	Enoxaparin	2
	ASA	1
VTE	No treatment	3
	Warfarin	2
	enoxaparin/fondaparinux	3
	ASA	0
Ischemic stroke	No treatment	3
	Warfarin	2
	Enoxaparin	0
	ASA	6

**Table 2:** Biochemical parameters detected before and after three months of direct oral anti-coagulant (DOAC) therapy. Continuous variables are expressed as mean ± SD; n.s (not statistically significant).

Parameter	Baseline	3 Months	*p*-Value
Hemoglobin (g/dL)	13.4 ± 1.7	13.1 ± 2.0	n.s.
Hematocrit, (%)	41 ± 5	40 ± 6	n.s.
Platelets, (10^9^/L)	209 ± 71	217 ± 65	n.s.
PT-INR	1.71 ± 0.65	1.27 ± 0.23	n.s.
aPTT, ratio	1.08 ± 0.20	1.18 ± 0.44	n.s.
Cholesterol, (mg/dL)	160 ± 30	162 ± 38	n.s.
HDL, (mg/dL)	52 ± 14	53 ± 14	n.s.
Triglycerides, (mg/dL)	111 ± 42	105 ± 30	n.s.
LDL, (mg/dL)	79 ± 24	83 ± 33	n.s.
Creatinine, (mg/dL)	1.09 ± 0.44	1.16 ± 0.45	n.s.
eGFR, (mL/min)	61 ± 22	57 ± 21	n.s.
AST, (U/L)	24 ± 14	19 ± 7	n.s.
ALT, (U/L)	23 ± 16	18 ± 8	n.s.
Calcium, (mg/dL)	8.7 ± 0.3	9.4 ± 0.4	n.s.
Vitamin D (25-OH), (ng/mL)	17 ± 7	25 ± 14	n.s.
CTX, (ng/mL)	0.31 ± 0.09	0.36 ± 0.12	<0.05

Note: *PT-INR*, prothrombin time-international normalized ratio; *aPTT*, activated partial thromboplastin time; *HDL*, high-density lipoproteins; *LDL*, low-density lipoproteins; *eGFR*, estimated glomerular filtration rate; *AST*, aspartate aminotransferase; *ALT*, alanine aminotransferase; *CTX*, C-telopeptide of type I collagen.

**Table 3:** Baseline PT-INR in patients classified according to the underlying disease.

Parameter	NVAF	VTE	Ischemic Stroke	*p* Value
PT-INR (baseline)	2.2 ± 0.5	1.5 ± 0.5	1.3 ± 0.3	<0.05

*PT-INR* prothrombin time-international normalized ratio, *NVAF* non-valvular atrial fibrillation, *VTE* venous thromboembolism.

**Table 4:** Cluster differentiation (CD) markers expression was similar in circulating progenitors harvested before and after three months of DOAC treatment. n.s (not statistically significant).

Cluster Differentiation Transcript	Pre DOACS	Post DOACS	*p*-Value
CD105	65 ± 0.6%	64% (±0.5)	n.s
CD 73	73% (±0.3)	72 (±0.2)	n.s
CD3	0%	0%	n.s
CD14	0.3% (±0.04)	0.4% (±0.06)	n.s
CD19	0%	0%	n.s
CD45	1.6% (±0.3)	1.8% (±0.4)	n.s
CD34	low level	low level	n.s
